# Assessment of Random Error in Phantom Dosimetry with the Use of Error Simulation in Statistical Software

**DOI:** 10.1155/2015/596858

**Published:** 2015-12-31

**Authors:** R. C. Hoogeveen, E. P. Martens, P. F. van der Stelt, W. E. R. Berkhout

**Affiliations:** ^1^Department of Oral and Maxillofacial Radiology, Academic Center for Dentistry Amsterdam (ACTA), Gustav Mahlerlaan 3004, 1081 LA Amsterdam, Netherlands; ^2^Statisticor, Statistical Research Office, Dorpsstraat 90, 2831 AT Gouderak, Netherlands

## Abstract

*Objective*. To investigate if software simulation is practical for quantifying random error (RE) in phantom dosimetry.* Materials and Methods.* We applied software error simulation to an existing dosimetry study. The specifications and the measurement values of this study were brought into the software (R version 3.0.2) together with the algorithm of the calculation of the effective dose (*E*). Four sources of RE were specified: (1) the calibration factor; (2) the background radiation correction; (3) the read-out process of the dosimeters; and (4) the fluctuation of the X-ray generator.* Results.* The amount of RE introduced by these sources was calculated on the basis of the experimental values and the mathematical rules of error propagation. The software repeated the calculations of *E* multiple times (*n* = 10,000) while attributing the applicable RE to the experimental values. A distribution of *E* emerged as a confidence interval around an expected value.* Conclusions.* Credible confidence intervals around *E* in phantom dose studies can be calculated by using software modelling of the experiment. With credible confidence intervals, the statistical significance of differences between protocols can be substantiated or rejected. This modelling software can also be used for a power analysis when planning phantom dose experiments.

## 1. Introduction 

When X-rays are used for diagnostic purposes, clinicians should follow the ALARA principle, which directs that the risk of radiation exposure to the patient should be* “As Low As Reasonably Achievable”* [1]. When different exposure protocols can be chosen to perform a diagnostic task it is therefore important to know for the clinician which protocol exposes the patient to the lowest dose of radiation. However, the effective dose (*E*) of an exposure cannot be measured directly. Instead, it must be calculated, and the International Committee on Radiation Protection (ICRP) has developed a system to calculate *E*. In maxillofacial radiology, *E* is frequently calculated by exposing an anthropomorphic phantom head, which contains dosimeters at specified locations, to radiation [2]. Using this method, the absorbed doses in different tissues and organs can be assessed, and these values can be used to calculate *E* by a weighted summation.

Like any experiment involving measurements, this method of calculating *E* is subject to error. Errors can be classified as systematic error and random error (RE). Systematic error can, for example, originate from incorrect calibration of the dosimeters or from uncertainties in the tissue weighting factors. Systematic errors are in nature not quantifiable because they are unknown. If measurement values reveal systematic errors then it is possible to eliminate them by compensating them. When research is conducted to compare different exposure protocols, systematic error only plays a limited role, since outcomes of dosimetry experiments of exposure protocols that are executed with the same equipment under the same conditions will all be influenced by the same systematic error(s) in the same way.

In contrast to systematic error, RE plays a role in comparative dosimetry because it influences the differences measured between protocols by inducing an artificial difference or masking or magnifying a true difference. RE can be regarded as a normal distribution of values around the assumed “true” value. RE in phantom dosimetry originates from a number of sources. These sources are inaccuracies in the calibration process; in the dose response of the dosimeters or their read-out process; in the correction of background radiation; or in the output of the X-ray generator. These different sources of RE propagate in the resulting inaccuracy of the calculated *E*, which as a result will also display a normal distribution.

Without a correct assessment of the size of RE, confidence intervals around the derived value of *E* cannot be calculated. Therefore, tests to substantiate the statistical significance of differences between protocols cannot be properly performed. In this case, the outcome of a study has limited value and statements about differences between protocols are not justified. Interestingly, comparative dosimetry studies have been published that provide absolute dose values without specifying a confidence interval [3, 4]. Only some of these publications provided an indication of the accuracy of the dosimeter system used and only a few quantified and incorporated the fluctuation of the output of the X-ray generator in the calculations. This fluctuation is an important source of RE, especially in protocols with low numbers of exposure cycles.

The calculation of *E* from energies detected in dosimeters after X-ray exposure follows a complex algorithm. The RE in the measured values propagates therefore in a complex way to the RE in the resulting *E*. The propagation of error cannot easily be calculated using the mathematical rules of error propagation because a number of variables that are sources of RE are dependent. For example, the read-out values of dosimeters of one location in the phantom head are used in calculating the tissue dose of different tissues. This dependency complicates a mathematical approach to quantifying RE of the resulting value of *E*.

One way to address and account for RE is to simulate the measurement process in software used for statistical calculations. This software could repeat the calculations of *E* while introducing RE around the measured values of the dosimetry experiments using Monte Carlo (MC) simulation. The extent of this artificially induced RE should be based on the specifications of the equipment used and/or the actual measurements acquired during the experiment. When multiple cycles (e.g., 10,000 cycles) of recalculation of the algorithm are repeated in the software with this RE simulation, a distribution of *E* will emerge. This would provide an *E* that is not a single value but, instead, is expressed as a confidence interval around an expected value, which would improve the relevance of the outcomes of dosimetry studies and would facilitate statements about the statistical significance of differences between protocols.

The aim of this paper was to investigate if software simulation is practical for quantifying error in phantom dosimetry. We applied software error simulation to an existing dosimetry study, using the original study's methods, equipment specifications, and results.

## 2. Methods

### 2.1. Dosimetry Experiment

To model a dosimetry study using the software simulation, we chose “Dose Reduction in Orthodontic Lateral Cephalography: Dosimetric Evaluation of a Novel Cephalographic Thyroid Protector (CTP) and Anatomical Cranial Collimator (ACC)” by Hoogeveen et al. as an example for this paper [5]. Briefly, this study involved lateral cephalographic exposures of a phantom head equipped with two thermoluminescent dosimeters (TLDs) at 25 specified locations. Four protocols with different shielding modalities of 50 exposures each (5 sec at 80 kV, 12 mA) were conducted. The TLDs were calibrated by exposing the phantom head to a defined dose, which was measured by a calibrated dosimeter, and then a mean calibration factor (CF) was calculated. The background radiation was corrected by leaving 3 calibrated TLDs unexposed during the protocol and subtracting their mean measured values from the read-out values of the exposed TLDs. The reading and annihilation of the TLDs were performed by a calibrated microprocessor-controlled oven. *E* of the 4 protocols was calculated according to the ICRP tissue weighting factors and the fractions of tissues exposed [2]. Finally, *E* was calculated per one cephalographic exposure of 0.6 sec.

### 2.2. Modelling the Experiment

All of the calculations for the dose experiment were programmed in open source software (R version 3.0.2; The R Foundation for Statistical Computing, Vienna, Austria; http://www.r-project.org/) and four components of the experiment were defined. First, the determination of the mean CF was modelled by dividing the average read-out values from the TLDs by the known calibration exposure dose. Second, the background correction was assessed by multiplying the CF by the average of the read-out values of the three TLDs that were unexposed. Third, the calculation of *E* from the read-out values of the TLDs was programmed by averaging the values of the two TLDs per location and compensating for background radiation. The different tissue doses were calculated by averaging the values of the corresponding locations and accounting for the irradiated fractions of the tissues. *E* was derived from a weighted summation of the tissue doses. Fourth, *E* for every protocol per exposure of 0.6 seconds was calculated by dividing the derived *E* by 416.7 (250 sec/0.6 sec), which was the ratio of the exposure dose of the phantom head in one protocol over the exposure dose of one cephalographic exposure.

### 2.3. Identifying and Quantifying RE

RE was introduced to the measurements in each of the 4 components of the experiment. For the software modelling, this RE was identified and quantified, and it was used in MC simulations to assess the RE of the resulting *E*. First, the RE of the CF was estimated to be the mean relative standard deviation (RSD)  of the individual CFs calculated for all TLDs. Instead of using a fixed mean value, RE was added to this value to perform the MC simulation. Second, the RE of the background radiation was estimated for each protocol separately using the RSD of the 3 values for the background radiation of the TLDs. Again, instead of using a fixed mean background value, RE was added to this value to perform the MC simulation. Third, the RE resulting from the TLD read-out process was derived from the measured values and was calculated to be the RSD of the 100 values of the TLD pairs (4 protocols with 25 locations each). It appeared that the RSD was negatively dependent on the read-out value: the higher the read-out value, the lower the RSD. Since the highest TLD values weigh heavily in the calculation of *E*, using an average value of the RSD would result in overestimation of the RSD of *E*. In the current dose study, this relation was best described by a quadratic function:

(1) RSD=4.57−1.98×10−7×value+3.29×10−15×value2,

where “value” is the number of pulses that is detected by the read-out oven. This formula was incorporated into the software to simulate RE around the TLD read-out values. Finally, the last RE in this experiment originated from the output of the X-ray generator used for the exposure. This RE of the output dose was previously tested with a calibrated dosimeter and estimated to have an RSD of 2.4%. In this experiment, a total of 50 exposures were performed per protocol, so the RE of the output was calculated using the mathematical rules of error propagation:

(2) $$\begin{array}{c} {\text{RSD}}_{\left(50\text{ exposures}\right)}=\frac{1}{50}\times \sqrt{50\times {\left({\text{RSD}}_{\left(1\text{ exposure}\right)}\right)}^{2}}. \end{array}$$

This RE was introduced into the model when *E* of a protocol of 250 sec of exposure was converted to *E* of 1 exposure of 0.6 sec.

## 3. Results

We created a software model to represent the steps needed to calculate *E* from the read-out values of the TLDs and we quantified the simulated RE in the 4 components of the experiment. First, the RE attributed to the CF was the RSD of the read-out value of the calibration exposure and was calculated to be 3.2%. Second, the RE of the background radiation correction was the RSD of the read-out values of the TLDs that were unexposed in each protocol. The REs for each of the 4 protocols were calculated to be 9.5%, 4.6%, 8.6%, and 1.3%. Third, the RSD of the TLD read-out system was defined by the quadratic equation in formula (1). The SD for the lowest TLD read-out value was 4.52% and the SD for the highest measured value was 1.67%. Fourth, the known RSD of the output of the X-ray generator was 2.4%. Using the rules of error propagation (formula (2)) for a protocol of 50 exposures, the SD was calculated to be 0.34%. Therefore, when converting to a single exposure, *E* of the protocol was divided by the simulated MC value using an RSD of 0.34% around 416.7.

**Figure 1:**
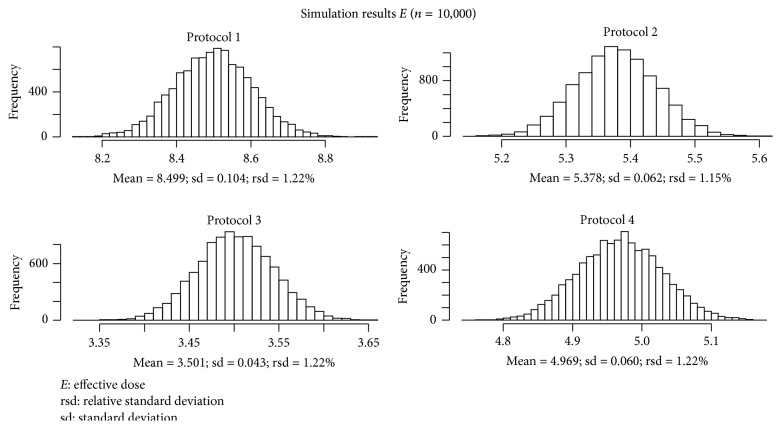
Graphic representation of the distribution of values of *E* in *μ*Sv after 10,000 calculation cycles.

**Figure 2:**
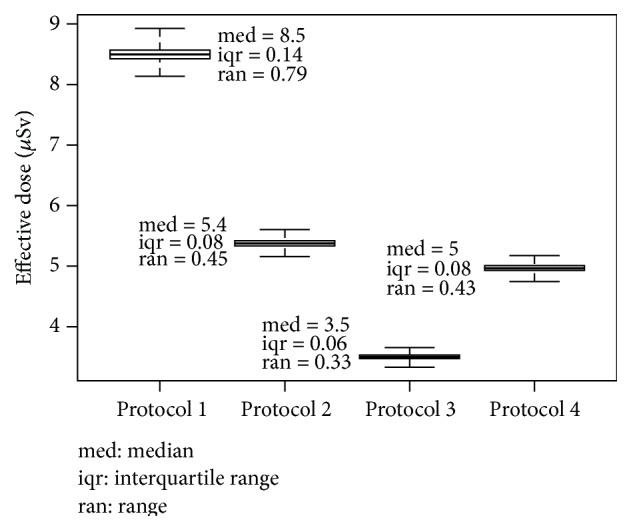
Box plots of the 4 protocols generated.

The software repeated the calculation of *E* 10,000 times for each protocol, using the measurements of the dose experiment and adding the applicable RE. The resulting values of *E* and their SDs and RSDs are shown in Figure 1. The RSD that emerged from the simulations ranged from 1.1% to 1.2%. The software also generated box plots for the resulting *E* according to the 4 protocols; these are shown in Figure 2.

## 4. Discussion

Dosimetry studies should include a confidence interval when presenting results. If no confidence interval is defined, the outcomes cannot be used to draw conclusions about the patient dose. Mathematical calculation of the propagation of error for dosimetry studies is complicated because data that contain RE are used multiple times in the calculations of *E*. In this model, we quantified RE in each step of the process and used simulation software to mimic the propagation of error generated in each step of the calculation of *E*; this proved a viable way to incorporate RE into the results. Using this method, a statistical test can be performed to assess the significance of differences between exposure protocols. This enables clinicians to make founded choices between exposure options.

To use this approach, the mimicking software must be programmed accurately. For example, the CF and its RE must be incorporated in the calculation in order to reflect its influence on the RE of *E*. In this study, the CF was only calculated once and then used for all 4 protocols. To assess the difference in *E* between protocols, the differences between protocols calculated with the same CF should be compared. Therefore, the software must measure the 4 protocols in 1 calculation cycle with 1 CF and register the differences. Otherwise, the RE of the differences between the protocols is overestimated.

The dose study used for this model was performed with TLDs, but this method of software modelling can also be applied to studies that use other types of dosimeters. When the precision of the specific systems used is incorporated in the simulation of RE, the multiple recalculations of *E* deliver an unbiased estimation of RE.

The dose study that was used as an example in this paper regarded a 2D imaging modality being cephalography. The method of assessing RE proposed in this paper can however be used for dose studies regarding all imaging modalities. The dosimeters in the phantom head record radiation at specified sites in the phantom head. Their accuracy of recording radiation is independent of the exposure modality that is deployed. In the modelling of the experiment however it is important to correctly incorporate the variation in output of the X-ray generator, which is dependent on the exposure modality.

The RE of the output of the X-ray generator used in the modelled experiment decreased from an RSD of 2.4% to an RSD of 0.34%. This change was due to the large number (*n* = 50) of exposures per protocol. If a small number of exposures had been used, the RE in the experiment would have been much larger. As mentioned previously, published dose studies often do not account for fluctuations in the output dose of the X-ray generator. In one study, a single cone beam computed tomography (CBCT) exposure of a phantom head was used and the stated RSD was less than 0.5%, which seems unrealistically low unless the device emits X-rays in a pulsed fashion and the stability of its output has been confirmed between consecutive cycles of use [3]. It is also important to note that the grey values of images of CBCT machines have a distinct pattern during consecutive cycles of use, although other explanations can be formulated; these fluctuations are possibly caused by changes in the output of the X-ray generator [6]. When a research protocol does not account for these changes, a systematic error can be introduced in the outcomes.

The simulation program modelled here can also be used in a power analysis when planning an experiment, such as deciding on the number of TLDs per location. If the dose study that served as an example for this model had been executed with one dosimeter per location instead of two, the RE in the final value of *E* would increase from an RSD of 1.1% to 1.2% to an RSD of 1.2% to 1.4%. If the expected differences between the protocols are small, the lower RSD would result in more power to reveal these differences. Also, the effect that different numbers of exposures per protocol would have on the RE can be assessed in advance. When the expected difference in *E* between protocols can be estimated in a planned dose study, the simulation program can be used for a power analysis of the research protocol.

Still, a reliable confidence interval around *E* does not mean that the use of *E* is a precise quantification of the absolute risk for an individual patient. In an editorial article in* Radiation Protection and Dosimetry*, Martin stated: “for a reference patient there is an uncertainty of ±40% for an 80–90% confidence limit for *E* as an indicator of the relative health risk for different medical procedures” [7]. In this same paper, he asserted that “*E* to a reference patient may be used during optimisation in radiology, when comparing doses from different techniques (…)” [7]. Thus, although uncertainties exist about the dose-risk relationship, *E* is an appropriate concept for the optimisation of X-ray practices when applied to groups of patients. When protocols are compared for optimisation purposes, statements about differences between protocols can only be made when a credible confidence interval is defined around the value of *E* for each of the protocols. The method is therefore relevant for clinical practice because it allows a more confident statement about comparison of *E* allowing a more educated choice between different exposure protocols. Our method is not aimed at providing a more confident statement about dose and risk for the individual patient.

In this paper, the term “Monte Carlo simulation” is applied in its original mathematical sense, and it refers to repeated calculations to approximate the probability of certain outcomes using random variables. In radiation dosimetry, the term “Monte Carlo simulation” is known as a method by which dosimetry simulations can be performed with dedicated software (e.g., PCXMC v. 2.0 software, STUK, Helsinki, Finland). Computer phantoms are radiated with simulated X-ray photons and on the basis of chances of interaction of the photons along their path, and along the paths of electrons and photons that are dislodged or created by the interactions, *E* of an exposure can be calculated. The mathematical technique used in the PCXMC software is Monte Carlo simulation. The dosimetry experiments described in this paper are experiments with real anthropomorphic phantoms with dosimeters radiated with real X-ray photons. The dosimetry experiments described in this paper are experiments with real anthropomorphic phantoms with dosimeters radiated with real X-ray photons. The computer phantoms that are used in virtual dose studies like those with the PCXMC software are anatomically not yet as detailed as physical phantoms. This is especially relevant when relatively small areas of the head and neck are irradiated as in dental and maxillofacial exposures and when collimation of specific parts of the beam is tested in dosimetric research. This underlines the continued relevance of phantom dose studies and with it the relevance of correct assessment of the confidence intervals around their results.

In conclusion, credible confidence intervals for derived *E* values in phantom dose studies can be calculated by using software modelling of the experiment that identifies, quantifies, and incorporates all sources of RE. With credible confidence intervals, the statistical significance of differences between protocols can be substantiated or rejected. This modelling software can also be used for a power analysis when planning phantom dose experiments.
